# Participants' evaluation of an internet-based group compassion-focused therapy program for young people in Sweden

**DOI:** 10.3389/fpsyg.2025.1548320

**Published:** 2025-03-25

**Authors:** Magnus Vestin, Jussi Jokinen, Ida Blomqvist, Inga Dennhag

**Affiliations:** ^1^Department of Clinical Sciences, Child and Adolescent Psychiatry, Umeå University, Umeå, Sweden; ^2^Department of Clinical Sciences, Psychiatry, Umeå University, Umeå, Sweden; ^3^Department of Clinical Neuroscience, Karolinska Institutet, Stockholm, Sweden

**Keywords:** compassion, young people, stress, depression, anxiety, group psychotherapy, internet-based psychotherapy

## Abstract

**Introduction:**

Online group compassion-focused therapy (CFT) has not been thoroughly studied in young people, and the participants' perspectives on the treatment are highly relevant.

**Methods:**

A seven-session internet-based group CFT program was evaluated for young people aged 15–20 (*n* = 42, females = 37). A self-report evaluation questionnaire, specifically designed for this study, was administered post-intervention. The current study investigated: (1) Potential factors influencing the treatment program goals (increase self-compassion, reduce self-criticism and increase affect-regulation abilities) and the participants' overall experience of the treatment; and (2) The effect each intervention exercise had on the program goals according to the participants' ratings.

**Results:**

Participants generally reported high satisfaction and had a high attendance rate. Only 2.4% of the participants completed less than half of the sessions and 71% of the participants attended at least six of the seven sessions. Exercises that integrated traditional cognitive-behavioral therapy (CBT) and Compassion-Focused Therapy (CFT) principles received high ratings, whereas breathing and mindfulness exercises were rated lower in many cases. Several compassion exercises were also highly rated, such as the *Threat-drive and security system*, based on CFT affect-regulation theory. The majority did not find home assignments helpful.

**Discussion:**

The findings suggest that internet-based group CFT could be an easily accessible and acceptable intervention for young people in primary care, though further research is warranted.

## 1 Introduction

In the past decades, there has been a significant increase globally in young people's emotional problems, especially among females (Bor et al., 2014; Shorey et al., 2022; Thapar et al., 2022). A systematic review and meta-analysis demonstrated a 1-year period prevalence of 8% and lifetime prevalence of 19% for adolescent major depressive disorder in Europe and North America (Shorey et al., 2022). The high prevalence and rising trend among young people reflect the current situation in Sweden (The Public Health Agency of Sweden, 2018; The Swedish National Board of Health and Welfare, 2024), where this study is conducted. In Sweden, the percentage of young adults (aged 15 to 29) experiencing symptoms of depression is higher than the European average (Hapke et al., 2019).

Young people often have several psychiatric conditions at the same time (described as comorbidity) (Kalin, 2020). The terms internalizing (e.g., depression and anxiety) and externalizing (e.g., impulsive behavior and substance abuse) are widely used based on the factor-analytically derived groupings of symptoms (Achenbach et al., 2016; American Psychiatric Association, 2013), indicating that there are benefits of transdiagnostic treatments (Brinckman et al., 2024). For example, mixed symptoms of anxiety and depression are more common than pure cases of these conditions (Weisz et al., 2023). By targeting shared underlying factors in depression, anxiety, and other emotional disorders, transdiagnostic treatments have broad applicability across diagnoses (Dalgleish et al., 2020; Steele et al., 2018). However, research in youth psychotherapy has shown weak results in effect size for depression (Weisz et al., 2023), which seems specifically low in this age group (Cuijpers et al., 2020), with a significantly negative trend over time (Weisz et al., 2019). At the same time, the results for anxiety are significantly better (Weisz et al., 2023), even though the psychotherapy content for depression and anxiety treatment overlaps (Chorpita and Daleiden, 2009).

One emerging transdiagnostic treatment is compassion-focused therapy (CFT), a third-wave cognitive behavioral therapy (CBT) (Perkins et al., 2023) targeting core psychological processes that are crucial for wellbeing (Gilbert and Simos, 2022). Adolescent depression is likely caused by a combination of biological vulnerabilities and environmental stressors, with identified risk factors including peer relationships, interpersonal difficulties, behavioral patterns, cognitive styles, emotional/temperamental traits, and biological factors (Hankin and Griffith, 2023). Instead of focusing on diagnostic symptoms, CFT aims to enhance self-compassion—the compassion individuals feel and show toward themselves—as an alternative to self-judgment, self-criticism, or shame, all of which are strongly linked to mental health problems such as depression (Bridge et al., 2023; Neff, 2003; Vidal and Soldevilla, 2023). Additionally, by cultivating self-compassion, individuals can activate the brain's “soothing system,” which supports emotional regulation (Gilbert, 2014). This may counteract heightened threat responses associated with biological vulnerabilities and environmental stressors, making CFT a promising approach for adolescents experiencing depression. CFT-based interventions may be especially important for young people, as they face challenges with social, academic, emotional and identity-building aspects of their lives (Tali et al., 2023).

CFT has not been thoroughly studied in young people; however, in adults CFT has been found to improve compassion-based outcomes and symptoms of depression, anxiety and stress (Craig et al., 2020; Kirby et al., 2017; Millard et al., 2023). Intervention studies performed with young people (e.g., Boggiss et al., 2020; Bratt et al., 2020; Karr et al., 2020; Seekis et al., 2023; Bridge et al., 2023) suggest positive outcomes similar to the adult studies. In correlation studies with young people, low self-compassion has a significant relationship with emotional problems, primarily depression, anxiety and stress (Bluth et al., 2016; Jonsson and Dennhag, 2023; Pullmer et al., 2019).

Psychotherapy research has primarily used quantitative measures of symptom change and function based on clinician report as the major source of information for primary outcomes (Krause et al., 2019). While results such as symptom change are important, only focusing on this kind of approach neglects the perspectives of the young participants (Bratt et al., 2020). The participants' perspectives on the treatment are highly relevant, specifically when investigating new treatments (Bratt et al., 2020; Grudin et al., 2023) and the use and effectiveness of CFT in young people is still an emerging area of research. The current treatment intervention was internet-based via a videoconferencing system, which further highlights the need for participants' views, as internet-based group interventions for young people is a relatively new form of setting.

Efforts are being made to try to identify mechanisms of change in psychotherapy, but the results for depression are sparse and mixed, and the understanding of what causes improvement in youth depression symptoms is limited (Weisz et al., 2023). For anxiety, the mechanisms of change have been shown to be more substantial (such as in the case of exposure) (Weisz et al., 2023). In the current study, one area of focus was on the participants' views regarding what was helpful, investigated with quantitative data in how they rated each exercise in terms of how well it helped them to reach the program goals.

The current study is part of a larger project in northern Sweden called CUST, Compassion-Focused Treatment for Young People with Stress (Dennhag, 2018), a project aimed at developing and studying internet-based compassion-focused interventions to reach young people with emotional problems in rural areas. We have previously completed an initial RCT (Vestin et al., 2025) in which the participating young people were randomized to a seven-session group internet-based CFT program via videoconferencing or a waitlist control.

### 1.1 Intervention

Details of the current intervention are provided in Vestin et al. (2025). Briefly, a new Swedish CFT workbook (Dennhag, 2021) inspired by Professor Paul Gilbert, Elaine Beaumont and Mary Welford's approach (Beaumont and Welford, 2020) was developed within the CUST project for young people in Sweden. The workbook consisted of seven core modules: (1) What is compassion?, (2) Understanding myself, (3) Life compass, (4) Self-compassion and my body, (5) Feelings and compassion, (6) Creating balanced thoughts, and (7) Imagination. The modules contain 20 exercises all together. During each session, theory and exercises were alternated and ended with an introduction for a home assignment. Each week, there were 2–3 different homework assignments, and participants could choose the one(s) most relevant to them. The group leaders reviewed the completed homework at the beginning of each session. The homework assignments were available in video and/or text formats, with content designed specifically for this age group.

The 7-week CFT program was conducted once a week as a 90-min group therapy session via videoconference, with a psychotherapist leading the treatment with the support of an assistant. Participants received instructions on how to log in using multi-factor authentication, along with a link for mobile access and reminders sent both the day before and on the day of the session. During the first session, they were provided with guidelines on creating a safe environment for the video conference sessions. They were also instructed to contact the leader or assistant if they experienced any technical issues.

The treatment program goals are to increase self-compassion, reduce self-criticism and increase affect-regulation abilities.

### 1.2 Aim

The current study aimed to investigate: (1) Potential factors influencing the treatment program goals (increase self-compassion, reduce self-criticism and increase affect-regulation abilities) and the participants' overall experience of the treatment according to the participants' ratings; and (2) The effect each intervention exercise had on the program goals of compassion, self-criticism and affect regulation according to the participants' ratings.

In summary, this study explores CFT, a promising yet underexplored treatment for young people (Egan et al., 2022; Tali et al., 2023). By cultivating self-compassion and reducing self-criticism (Gilbert, 2014; Gilbert and Simos, 2022), CFT addresses core issues that differ from the primary focus of other therapies. CFT offers unique benefits for young people by emphasizing the development of self-compassion and emotional regulation (Tali et al., 2023). Delivering CFT in an online group format provides access to young people in remote or sparsely populated areas who might otherwise lack treatment opportunities. The current study investigates participants' views on the exercises, other factors, and their frequency of participation. To our knowledge, participants' ratings of the exercises have not been explored in previous CFT studies with this population. This could contribute to the understanding of what helps them achieve the CFT program goals.

## 2 Methods

### 2.1 Study design

This is a quantitative evaluation of the CFT program. It is part of the CUST project (Compassion-Focused Therapy for Young People with Stress) in northern Sweden (Dennhag, 2018), which aims to develop and evaluate internet-delivered compassion-focused interventions for young people with emotional problems.

A self-report evaluation questionnaire, specifically designed for this study, was conducted after the seven-session CFT program. In the beginning of the evaluation, a summary of the treatment sessions was communicated both visually and verbally as a reminder. The participants then responded independently to an online questionnaire with questions about the treatment, where they also rated each exercise separately regarding whether the specific intervention had any effect on compassion, self-criticism and affect regulation.

We analyzed the results that answered the study aim, but also a few crucial questions including satisfaction with the CFT program, if the training helped the participants to deal with their problems in a better way, and the importance of the treatment.

### 2.2 Ethics

This study was approved by the Swedish Ethical Review Authority (no. 2021-04357; 2022-01318-02; 2022-02931-02). Informed consents were obtained for all participants. The participants had information about the evaluation questionnaire beforehand and were informed that it was voluntary.

### 2.3 Procedure

Young people were informed about the CFT program study through social media and flyers, and through personal contact by staff or individuals included in this project at schools and primary-care health centers in northern Sweden. Inclusion criteria for participation required participants to be aged 15 to 20, to have self-perceived stress, anxiety, and/or depression, ability to speak and read Swedish, having at least one close, stable relationship with an adult, and the capacity to participate in an online group setting. All young people who participated in the CFT program for at least one session from 2022 to 2024 were asked to complete the evaluation questionnaire after completed treatment. The current study included RCT study participants, participants that received treatment after waitlist, and participants who had fewer symptoms than cut-off for the RCT study. All participants were relevant for evaluating the treatment's acceptability and usefulness. The evaluation questionnaire designed specifically for this study was completed 1 week after the last session.

### 2.4 Participants

Descriptives are presented in Table 1. There were *N* = 56 young people who attended at least one session in the CFT program and *N* = 43 of these completed the questionnaire developed for this current study. Of those, one person only participated in the last session and was therefore excluded. One participant turned 21 before the pre-assessments and treatment started but was included in the analyses. In the end, *N* = 42 young people, *n* = 37 females and *n* = 5 males, with a median age of 17.5 years (15 to 21 years) were included in the analyses.

**Table 1:** Descriptive statistics of the sample.

	**N (%)**	**M**	**SD**
**Sex**			
Female	37 (88.1)		
Male	5 (11.9)		
**Age**		**17.62**	**1.81**
15	7 (16.7)		
16	6 (14.3)		
17	8 (19.0)		
18	5 (11.9)		
19	8 (19.0)		
20	7 (16.7)		
21	1 (2.4)		
**Socioeconomic classification of parent(s)/caregiver(s)**			
Unemployed or on sick leave	1 (2.4)		
Students	1 (2.4)		
Workers	4 (9.5)		
Assistants and intermediate non-manual workers	17 (40.5)		
Professionals, civil servants, and executives	19 (45.2)		
**Living**			
With parents/parent	29 (69.0)		
Alone	6 (14.3)		
Other/not specified	7 (16.7)		
**Self-report measures**			
TSCC Anxiety	41 (97.6)	8.29	4.44
PSS-10	39 (92.9)	21.97	5.56
MADRS-Y	41 (97.6)	22.59	10.23
CEASY-SE Self-compassion	37 (88.1)	51.11	15.42
CEASY-SE Compassion for others	37 (88.1)	75.76	11.66
CEASY-SE Compassion from others	37 (88.1)	58.78	16.05

*N* = 42. PSS-10, The 10-item Perceived Stress Scale; TSCC Anxiety, the subscale Anxiety from Trauma Symptom Checklist for Children; CEASY-SE Self-compassion, the subscale Self-compassion from The Compassionate Engagement and Action Scale for Adolescents; CEASY-SE Compassion for others, the subscale For others from The Compassionate Engagement and Action Scale for Adolescents; CEASY-SE Compassion from others, The subscale From others from The Compassionate Engagement and Action Scale for Adolescents; MADRS-Y, Montgomery-Åsberg Depression Rating Scale-Youth.

### 2.5 Measure

All measures are self-reported questionnaires.

#### 2.5.1 Description of the evaluation questionnaire designed for this study

An evaluation self-report questionnaire was designed specifically for this study to gather data about the treatment effect on the program goals regarding compassion, self-criticism and affect-regulation abilities, and to investigate how helpful or not each exercise was in fulfilling the treatment goal. In the first four items, the participants rated potential contributors to the treatment program goals such as homework and course material. They were then asked to rate the 20 exercises regarding if they had been helpful in reaching the program goals using a 5-point scale: 0 (No, not at all), 1 (Yes, a little), 2 (Yes, sufficiently), 3 (Yes, more than sufficiently), and 4 (Yes, to a large extent). Furthermore, they were asked to select the three exercises of the first ten that they felt contributed the most regarding reaching the program goals to increase self-compassion, reduce self-criticism and increase affect-regulation abilities. They were then posed the same question about selecting the three exercises that contributed the most to reaching the program goals, but for the last ten exercises. There were limitations in the system we used (Microsoft FORMS) that forced us to split the 20 exercises into two blocks (1–10 and 11–20) for the ratings of the top three exercises as described above. Finally, the questionnaire contained seven dimensional questions about the participant's perspective with a focus on their experience of the treatment, whether they would recommend the treatment to a friend in need of similar help, and how helpful the treatment was in coping with their problems. The questionnaire is available as Supplementary material.

#### 2.5.2 Measures to assess symptoms of stress, depression, anxiety and compassion pre-treatment

##### 2.5.2.1 Trauma Symptom Checklist for Children (TSCC)

TSCC (Briere, 1996) is a self-report questionnaire for young people 8–17 years old measuring symptoms of traumatic experiences. The self-report is measured with a 4-point scale ranging from 0 (“never”) to 3 (“almost all the time”). The internal consistency has been shown to be good (Briere, 1996), including for the Swedish translation (Nilsson et al., 2008). TSCC has six clinical scales, and the current study used the clinical scale Anxiety, which includes nine items. In the current study, the Cronbach's alpha was 0.88.

##### 2.5.2.2 The 10-item Perceived Stress Scale (PSS-10)

PSS-10 (Cohen, 1988) is a self-report questionnaire for young people and adults measuring feelings and thoughts regarding stress-related components. The self-report is measured with a 5-point scale, ranging from 0 to 4, and a higher score indicates higher stress level (Nordin and Nordin, 2013). The PSS-10 has acceptable psychometric properties (Lee, 2012) and the internal consistency has been shown to be good for the Swedish translation (Nordin and Nordin, 2013). In the current study, the Cronbach's alpha was 0.83.

##### 2.5.2.3 Montgomery–Åsberg Depression Rating Scale – Youth (MADRS-Y)

MADRS-Y is a self-report questionnaire for young people 12–22 years old (Vestin et al., 2024, 2022) adapted from the adult version MADRS-S (Svanborg and Åsberg, 1994). The 12-item self-report is measured with a 7-point scale ranging from 0 (low) to 6 (high), and a higher score indicates more severe depression. The internal consistency has been shown to be good in Swedish samples (Vestin et al., 2024, 2022). In the current study, the Cronbach's alpha was 0.84.

##### 2.5.2.4 Compassionate Engagement and Action Scales for Youths – Swedish version (CEASY – SE)

CEASY-SE is a self-report questionnaire for adolescents adapted from the adult version CEAS (Gilbert et al., 2017) and tailored to the Swedish context, including the three subscales compassion for others, compassion from others, and self-compassion (Henje et al., 2020). The 30-item self-report is measured with a 10-point scale ranging from 1 (“never”) to 10 (“always”). The three control items are removed in this study, with 27 items remaining. Internal consistency has been shown to be good in a Swedish sample (Henje et al., 2020). In the current study, the subscales are analyzed separately, and the Cronbach's alpha was 0.92 for compassion for others, 0.90 for compassion from others and 0.89 for self-compassion.

### 2.6 Statistical analysis

All analyses were carried out using IBM SPSS Statistics version 29.0. Descriptive statistics were used to describe the participants and for the analyses of the evaluation questionnaire developed for this study. Multiple imputations were performed due to 9.92 percent missing data in the self-report measures. To investigate the level of depressive symptoms in the sample, the MADRS-Y questionnaire mean was compared with the mean from two normative samples (Vestin et al., 2022) using the one-sample *t*-test.

## 3 Results

Regarding the internet-based CFT group session attendance, almost 98 percent attended at least four of the seven sessions, and about 71 percent attended at least six of the seven sessions, as seen in Table 2.

**Table 2:** Number of completed sessions during treatment.

**Number of completed sessions**	**N completed n = 42**	**Percent %**
2 sessions	1	2.4
3 sessions	0	0.0
4 sessions	7	16.7
5 sessions	4	9.5
6 sessions	19	45.2
7 sessions	11	26.2

### 3.1 The CFT program goals

Most of the participants experienced that the content and goals of the treatment were made clear before the start of the interventions (Yes = 85% and Somewhat = 11.9%), and that it had been addressed during the interventions (Yes = 73.8% and Somewhat = 23.8%). Regarding whether the home assignments helped in reaching the goals, the majority did not rate that it had helped sufficiently, 47.6 percent rated “Yes a little” and 9.5 percent “Cannot answer because I have not done the home assignments or other reason.” Participants' ratings regarding the program goals are presented in Table 3.

**Table 3:** Participants' ratings regarding the treatment goals: increase self-compassion, reduce self-criticism and increase affect regulation strategies.

Were the content and goals of the treatment made clear before the start of training?					
Yes	Somewhat	No	Cannot answer		
85.7%	11.9%	2.4%	0%		
**Do you feel that what was stated in the goals has been addressed in the CFT training?**					
Yes	Somewhat	No	Cannot answer		
73.8%	23.8%	2.4%	0%		
**Do you feel that the course materials (PDF of Powerpoint and PDF of the manual) have helped you reach your goals?**					
Yes, absolutely excellent assistance	Yes, more than sufficiently	Yes, sufficiently	Yes, a little	No, not at all	Cannot answer
4.8%	19.0%	33.3%	33.3%	4.8%	4.8%
**Do you feel that the homework assignments have helped you reach your goals?**					
Yes, absolutely excellent assistance	Yes, more than sufficiently	Yes, sufficiently	Yes, a little	No, not at all	Cannot answer
2.4%	11.9%	28.6%	47.6%	0.0%	9.5%

*N* = 42.

### 3.2 Evaluation of the exercises regarding the CFT program goals

The mean group scores for the exercises were calculated. Overall, the mean shows that all exercises were rated as being helpful to reach the program goals to some extent (M = 1, “Yes, a little,” or higher) except for exercise 11) *Breathe – balloon* (M = 0.97). Eleven of the 20 exercises had a mean of 2, “Yes, sufficiently,” or higher. The top three highest ratings were for the exercises 14) *Do an analysis of your emotions – vulnerability, trigger, feeling, thought and behavior* (M = 2.39), 18) *Create a safe place* (M = 2.32) and 6) *What do you value highly in life?* (M = 2.28). Table 4 shows the mean value, standard deviation and ratings in percentage for each statement for each exercise.

**Table 4:** Participants ratings of the exercises in the modules.

		**Item labels % (n)**						**Mean (SD)**
	Question to the participants: Do you think that the exercises below have helped you achieve the goals (increase self-compassion, reduce self-criticism and increase affect regulation skills)? Exercise	No, not at all.	Yes, a little.	Yes, good enough.	Yes, more than good.	Yes, to a large extent.	I wasn't there/Don't remember.	Of the group score (0 not at all – 4 Yes, to a large extent).
Module 1. What is compassion.	1. The threat-drive and security system.	4.8 (2)	38.1 (16)	19.0 (8)	16.7 (7)	19.0 (8)	2.4 (1)	2.07 (1.25)
	2. Breathe with focus on you.	9.5 (4)	23.8 (10)	23.8 (10)	19.0 (8)	16.7 (7)	7.1 (3)	2.10 (1.27)
Module 2. Understanding myself.	3. The train - imagine going back in time by train. What has influenced you in life?	14.3 (6)	19.0 (8)	21.4 (9)	26.2 (11)	11.9 (5)	7.1 (3)	2.03 (1.29)
	4. Try to understand yourself.	4.8 (2)	23.8 (10)	31.0 (13)	19.0 (8)	16.7 (7)	4.8 (2)	2.20 (1.16)
	5. The healing ray of light.	7.1 (3)	50.0 (21)	9.5 (4)	16.7 (7)	2.4 (1)	14.3 (6)	1.50 (1.00)
Module 3. Direction in life.	6. What do you value highly in life.	4.8 (2)	14.3 (6)	31.0 (13)	23.8 (10)	11.9 (5)	14.3 (6)	**2.28** (1.09)
	7. Create an image of compassion.	9.5 (4)	40.5 (17)	16.7 (7)	14.3 (6)	4.8 (2)	14.3 (6)	1.58 (1.08)
	8. Draw a tree of life.	16.7 (7)	35.7 (15)	19.0 (8)	4.8 (2)	14.3 (6)	9.5 (4)	1.61 (1.31)
Module 4. Be kind to myself about my body.	9. Personal goals regarding food, sleep, and activity.	9.5 (4)	19.0 (8)	23.8 (10)	16.8 (7)	14.3 (6)	16.7 (7)	2.09 (1.27)
	10. Switch seats – imagine that you are talking to yourself with a friendly tone.	7.1 (3)	31.0 (13)	19.0 (8)	7.1 (3)	19.0 (8)	16.7 (7)	2.00 (1.33)
	11. Breathe – balloon.	16.7 (7)	40.5 (17)	14.3 (6)	0.0 (0)	0.0 (0)	28.6 (12)	**0.97** (0.67)
	12. Breathe – square.	16.7 (7)	33.3 (14)	26.2 (11)	9.5 (4)	4.8 (2)	9.5 (4)	1.47 (1.08)
	13. Body control – stand up and close your eyes and imagine a push.	33.3 (14)	28.6 (12)	16.7 (7)	4.8 (2)	2.4 (1)	14.3 (6)	**1.00** (1.04)
Module 5. Emotions and compassion.	14. Do an analysis of your emotions - vulnerability, trigger, feeling, thought and behavior.	2.4 (1)	21.4 (9)	23.8 (10)	23.8 (10)	19.0 (8)	9.5 (4)	**2.39** (1.15)
	15. Mountain and stable – a mindfulness practice.	14.3 (6)	26.3 (11)	14.3 (6)	11.9 (5)	0.0 (0)	33.3 (14)	**1.36** (1.03)
Module 6. Create Balanced thoughts.	16. Identify thought traps and create alternative thoughts.	4.8 (2)	23.8 (10)	23.8 (10)	21.4 (9)	19.0 (8)	7.1 (3)	2.28 (1.21)
	17. How is your tone? Practice talking to yourself in a friendly tone.	11.9 (5)	28.6 (12)	16.7 (7)	7.1 (3)	9.5 (4)	26.2 (11)	1.65 (1.25)
Module 7. Use your imagination.	18. Create a safe place.	4.8 (2)	26.2 (11)	16.7 (7)	21.4 (9)	21.4 (9)	9.5 (4)	**2.32** (1.28)
	19. Make a mind map of things that speaks against your self-critical voice.	7.1 (3)	19.0 (8)	21.4 (9)	16.7 (7)	7.1 (3)	28.6 (12)	1.97 (1.16)
	20. Imagination and music. Think about something stressful, listen to music, relax.	7.01(3)	23.8 (10)	23.8 (10)	14.3 (6)	14.3 (6)	16.7 (7)	2.06 (1.24)

*N* = 42. Means highlighted in bold indicate the three highest and three lowest values, reflecting those items perceived as most and least helpful in achieving treatment goals.

**Figure 1:**
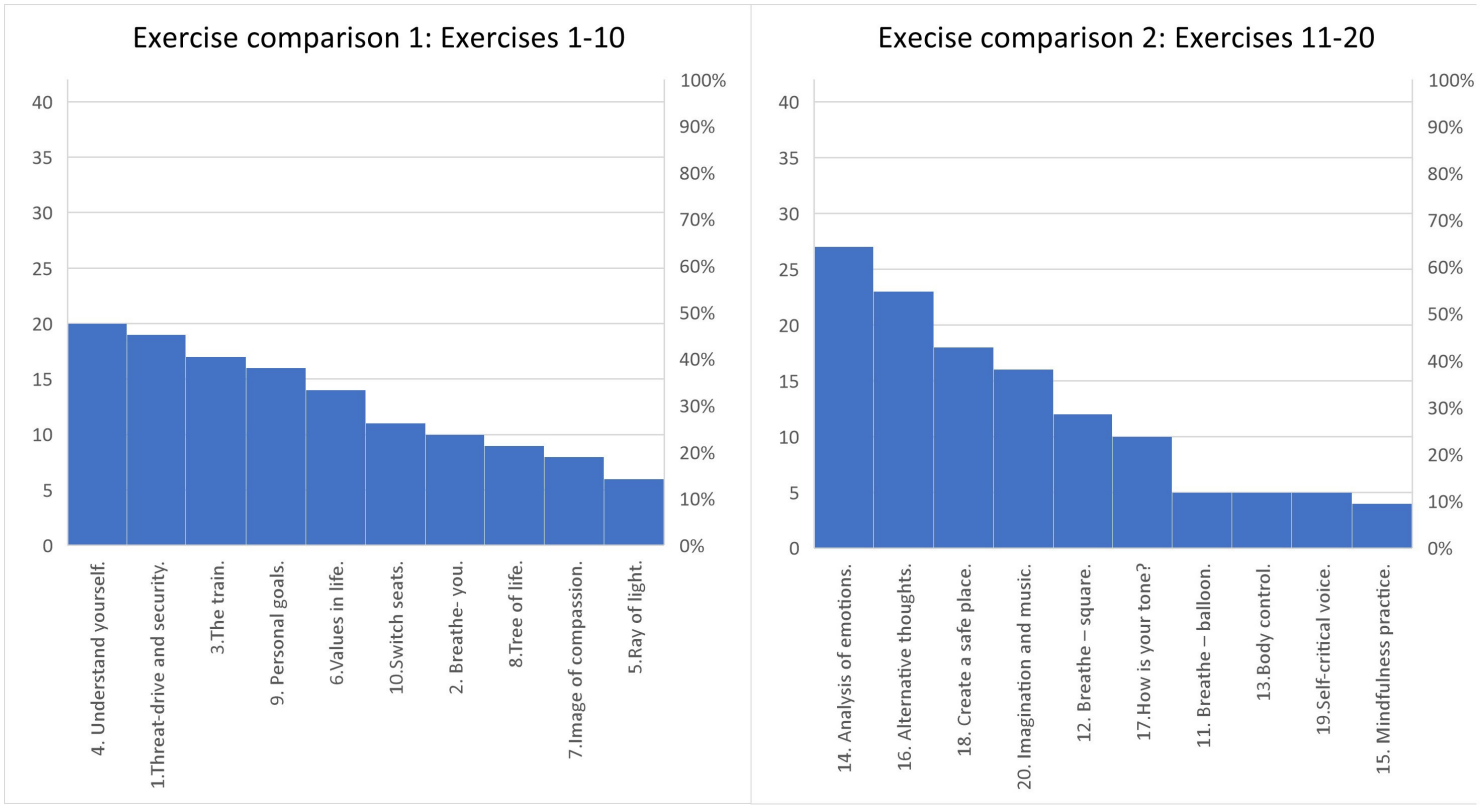
Participants' rating of the most helpful exercises. *N* = 42. The participants rated the top 3 most helpful exercises of the first 10 (1-10). Then they were asked to rate the top 3 most helpful exercises of the last 10 (11-20). Totally they rated 6 exercises as most helpful of 20. The result is presented with the highest ratings starting from the left for exercise 1-10 and then for 11-20. The percentage is presented to visualize the proportion of how many that rated each exercise as top 3. The number before each exercise represents the order in which the exercise was conducted.

Furthermore, the participants rated the top three of the first ten exercises (comparison one) and the top three of the last ten exercises (comparison two) regarding how helpful the exercises were in reaching the goals. As shown in Figure 1, the most popular three exercises in comparison one were *Try to understand yourself* , *The threat-drive and security system*, and *The train – imagine going back in time by train*. The most popular three exercises in comparison two were *Do an analysis of your emotions – vulnerability, trigger, feeling, thought and behavior, Identify distorted thoughts – create alternative thoughts*, and *Create a safe place*.

### 3.3 Satisfaction, problem-dealing and importance

Regarding satisfaction concerning the CFT program as a whole, 35.7 percent were “very satisfied,” 45.2 percent “mostly satisfied” and 19.0 percent were “neither satisfied nor dissatisfied.” None of the participants were “dissatisfied.” Regarding whether the training helped the participants to deal with their problems in a better way, 26.2 percent answered “Yes, it helped a lot,” 61.9 percent “Yes, it has helped to some extent,” 11.9 percent “No, it hasn't helped,” and 0.0 percent “No, it seems to have made it worse.” Regarding whether the compassion treatment had an importance for the participants, 21.4 percent answered “Yes, always,” 42.9 percent “Yes, most of the time,” 28.6 percent “Yes, to some extent,” 7.1 percent “Yes, at some point” and 0.0 percent “No, not at all.”

### 3.4 Comparison of depression symptoms with a normative sample

The mean MADRS-Y depression score was compared with the score from a MADRS-Y normative study (Vestin et al., 2022) with students 12–20 years old. The MADRS-Y study had two normative samples (*N* = 310 in each, with *n* = 187 females in sample one and *n* = 196 females in sample two). The mean total score was 11.39 (SD = 10.18) for sample one and 12.33 (SD = 10.66) for sample two. The current sample had significantly higher total scores at pretest (M = 22.72, SD = 11.16) than sample one [*t*_(41)_ = 6.58, *p* < 0.001 two-tailed] and sample two [*t*_(41)_ = 6.03, *p* < 0.001 two-tailed].

## 4 Discussion

The analyses included 37 females and 5 males, reflecting the clinical reality and the well-documented gender disparity observed in depression diagnoses. More females than males are affected, with particularly stark gender differences for adolescents and young women (Choate, 2020). Future research could explore strategies to increase male participation in similar interventions. This may involve tailoring recruitment strategies and examining potential gender-specific barriers to engagement. Given the uneven gender distribution in the sample, the findings may be more representative of young females, and the results should be interpreted with caution when applied to a broader population. According to the self-reported depression measure MADRS-Y, the sample had higher scores on depression in comparison with the normative population (Vestin et al., 2022).

Regarding adherence, 71% of the participants attended at least six of the seven sessions. The present study thus demonstrated a high rate of attendance, which indicates that the intervention is an acceptable treatment alternative. In a previous study by Grudin et al. (2022), participants who completed less than half of the modules were considered to have discontinued the treatment. In the present study, this accounted for only 2.4% of participants.

### 4.1 The CFT program goals

The participants' ratings suggest the program goals were made clear before the start of the interventions and addressed during the CFT program. The majority rated that they had a little or sufficient help to reach the goals from the files, including the manual and the presentation material, which indicates that the material was used and the content helpful to some extent, although it was not perceived as excellent assistance.

The majority did not rate that homework had helped them sufficiently to reach the program goals, despite the established role of homework as a crucial component of CBT, at least for adults (Kazantzis et al., 2004), where it is often demonstrated that homework and therapeutic outcome have a robust positive association (Farmer and Chapman, 2008). The impact of homework for adolescents or young people is not as clear and includes different aspects than for adults (Hildebrand-Burke et al., 2024), such as perceived additional academic responsibilities, which is challenging. A qualitative study involving depressed adolescents experiencing fatigue demonstrated that homework was perceived as demanding, with one participant, for example, stating “It was making me feel worse” (Herring et al., 2022). In the current study, most of the participants were students with additional academic responsibilities and with internalizing symptoms. These factors, as discussed, make completing homework more difficult and may reduce its perceived importance in the treatment process. The lack of personalized content in the homework assignments could also have influenced the effect. Other client or therapist factors such as client–therapist collaboration, and therapist involvement (e.g., detailed rationale, reinforcement, and exploring doubts and obstacles) (Farmer and Chapman, 2008; Hildebrand-Burke et al., 2024) could be other explanations for the result regarding homework, but was not investigated in the current study. Future studies could evaluate whether tailoring homework assignments to individual needs enhances effectiveness.

### 4.2 Evaluation of the exercises regarding the CFT program goals

The mean value of the participants' ratings indicated that the exercises were helpful in reaching the program goals. Eleven of the 20 exercises had a mean of 2, “Yes, sufficiently,” or higher, which shows that many of the exercises had a perceived effect for the participants. Considering that the intervention format was brief and accessible, this insight is promising.

A pattern is seen in which interventions related to traditional CBT are highly rated, with the exercise *Do an analysis of your emotions – vulnerability, trigger, feeling, thought and behavior* as the highest rated regarding helpfulness in reaching the goals, while breathing exercises as well as mindfulness exercises in many cases had lower ratings, with the exercise *Breathe – balloon* having the lowest rating. Mindfulness-based interventions for young people have in clinical settings outperformed treatment as usual for self-reported internalizing symptoms (Biegel et al., 2009). At the same time, some people can perceive mindfulness and breathing practice as boring and pointless and others as producing too many emotions (Burrows, 2022). In the current study, we suggest that interventions related to traditional CBT may have been more effective in aligning with the CFT program goals, providing a clearer and more meaningful approach for the participants. The fact that *Do an analysis of your emotions – vulnerability, trigger, feeling, thought and behavior* had the highest ranking, supports the importance of cognitive behavioral analysis in treatments targeting self-compassion, self-criticism and affect-regulation abilities.

However, there were also compassion exercises that contributed to reaching the program goals. When the participants rated the top three of the first ten exercises, all three had CFT content. These three: *Try to understand yourself*, *The threat-drive and security system*, and *The train – imagine going back in time by train*, were also rated as sufficiently helpful in reaching the goals. This indicates that certain compassion-focused exercises played a significant role in helping participants to achieve the program goals. *The threat-drive and security system* exercise is based on the theory CFT uses regarding affect regulation: the drive system, which focuses on pursuing and activating engagement strategies; the threat system, which is focused on detecting and responding to threats; and the soothing system, a positive system for well-being that helps regulate the threat response (Gilbert, 2014). *The train – imagine going back in time by train* is a newly developed exercise for the current CFT program and appears to have worked well in a group setting, bringing up stressful events in a respectful manner.

### 4.3 Internet-based group treatment

The treatment was internet-based, using a videoconferencing system to increase accessibility. Evidence from research indicates that internet-based interventions such as videoconferencing are effective in helping young people with their mental health (Karing, 2023; Porter et al., 2022; Stewart et al., 2017; von Wirth et al., 2023; Välimäki et al., 2017). Additionally, it is suggested that these interventions may also benefit socioeconomically and digitally marginalized young people (Piers et al., 2023). Providing internet-based group CFT appears to be an acceptable treatment alternative, as indicated by overall satisfaction with the treatment and high attendance. However, conclusions about the online format remain speculative, as participants were not specifically asked about the internet-delivered aspect of the treatment.

Group therapy has advantages, such as the ability to help several young people at the same time and for the participants to learn from each other, but there are also challenges, such as difficulties for some to participate in this kind of setting (Erekson et al., 2024). It has been suggested that the effects of psychotherapy for young people in group vs. individual therapy are similar (Eckshtain et al., 2020). Overall, the participants in the current study reported satisfaction with the treatment. However, it is important to consider that the participants actively contacted us to participate, and to some extent were open to receiving treatment in a group setting.

### 4.4 Limitations

The modules were given in the same order to everyone, and the evaluation questionnaire was carried out 1 week after the group had completed all the exercises, and memory bias can therefore be expected. Another option would be to use a design that allows measures every week at the end of the module instead. However, the advantage of our design is that participants can reflect over the contribution of a single exercise in the context of the entire treatment at the end of the program.

The study used an internet-based approach but did not specifically evaluate the online format or participants' engagement (such as participation in discussions, commitment to completing exercises, or personal reflections). However, due to the study's design and scope, the focus was on the intervention itself rather than the medium through which it was delivered. Future research would benefit from investigating these factors via, for example, participant experience surveys.

While this study relies on self-report measures and does not include methodological triangulation, such as in-depth interviews or observations, we acknowledge the value of such approaches. This quantitative design is limited, as it relies almost exclusively on self-report measures (and the number of completed sessions during treatment), which can introduce bias. Additionally, it does not allow for in-depth understanding, which is important in this research stage. For example, we found that the manual and PowerPoint files were helpful to some extent, but not which content, in what way it was helpful, or how it could be improved to be perceived as an excellent source of help in reaching the goals. Adding a qualitative interview study would provide valuable insights and deepen the understanding of participants' experiences. The strength of the current approach, which can be seen as complementary to the RCT study (Vestin et al., 2025) and a planned qualitative study, is that the answers can be quantitatively measured, and be helpful in the investigation of which of the exercises are the most appropriate to use to reach the program goals.

Addressing these limitations will be important for the successful implementation of online group CFT in mental health services for young people in sparse and remote areas. For example, our sample primarily consisted of female participants, which may limit the applicability of the findings to male adolescents. Furthermore, implementing online group CFT requires additional considerations, including training practitioners in this specific approach and ensuring consistency in therapy delivery.

### 4.5 Implications

Finally, studies investigating the participants' perspectives could provide valuable insights into how the young people themselves perceive the intervention, and what is helpful for them and what is not. This is crucial in psychotherapy research. In the current study, the participants generally reported satisfaction with the treatment and had high attendance rates. Adding that the treatment is both cost-effective and easily accessible further suggests that this internet-based group CFT program for young people could be a suitable and effective method in primary care settings.

## Data Availability

The data supporting the findings of this study are available from the corresponding author, Magnus Vestin, upon reasonable request.
