# Supplementary material for: Participants' evaluation of an internet-based group compassion-focused therapy program for young people in Sweden
Source: Front Psychol. 2025 Mar 25;16:1548320. doi: 10.3389/fpsyg.2025.1548320 (PMC11975948; doi:10.3389/fpsyg.2025.1548320)
Supplement: Supplementary file 1 [file Supplementary_file_1.docx]

| CUST evaluation |
| --- |
| Please help us improve our Compassion-Focused Training (CFT) by answering a few questions about the training you received. We are interested in your honest answers, regardless of whether they are positive or negative. The survey will take about 6 minutes. Thank you very much! We really appreciate your help!  1. Were the content and goals of the treatment made clear before the start of training? The goals were to increase self-compassion, to reduce self-criticism, and to be better at regulating uncomfortable feelings. |
| Yes |
| Somewhat |
| No  Cannot answer |
| 2. Do you feel that what was stated in the goals has been addressed in the CFT training? |
| Yes |
| Somewhat |
| No  Cannot answer  3. Do you feel that the course materials (PDF of PP and PDF of book) have helped you reach your goals (increase self-compassion, reduce self-criticism and increase affect-regulation strategies)?  No, not at all  Yes, a little  Yes, sufficiently  Yes, more than sufficiently  Yes, absolutely excellent assistance  I cannot answer because I have not read or used the materials  4. Do you feel that the homework assignments have helped you reach your goals (increase self-compassion, reduce self-criticism and increase affect-regulation strategies)?  No, not at all  Yes, a little  Yes, sufficiently  Yes, more than sufficiently  Yes, absolutely excellent assistance  I cannot answer because I have not done the homework assignments  5. Do you feel that the following exercises have helped you reach your goals (increase self-compassion, reduce self-criticism and increase affect-regulation abilities)?   \|  \| No, not at all \| Yes, a little \| Yes, sufficiently \| Yes, more than sufficiently \| Yes, to a large extent \| Did not do/No \| \| --- \| --- \| --- \| --- \| --- \| --- \| --- \| \| Ex. 1: The threat-drive and security system- Draw what you have in the circles \|  \|  \|  \|  \|  \|  \| \| Ex. 2: Self-focused breathing – mindfulness exercise \|  \|  \|  \|  \|  \|  \| \| Ex. 3: The train \|  \|  \|  \|  \|  \|  \| \| Ex. 4: Try to understand yourself – fill in background, problems, strategies, etc. \|  \|  \|  \|  \|  \|  \| \| Ex. 5: Healing ray of light – mindfulness exercise focused on tension/fatigue \|  \|  \|  \|  \|  \|  \| \| Ex. 6: What do you value highly in your life? Choose 5 values. \|  \|  \|  \|  \|  \|  \| \| Ex. 7: Create an image of compassion \|  \|  \|  \|  \|  \|  \| \| Ex. 8: Draw a tree of life \|  \|  \|  \|  \|  \|  \| \| Ex. 9: Personal goals regarding food, drink, sleep, activities \|  \|  \|  \|  \|  \|  \| \| Ex. 10: Switch seats – imagine that you are talking to yourself with a friendly tone. \|  \|  \|  \|  \|  \|  \| \|  \|  \|  \|  \|  \|  \|  \|   6. Rate the exercises. Mark only three (3) of the exercises – the exercises that you think most helped you reach your goals (increase self-compassion, reduce self-criticism and increase affect-regulation abilities).   \|  \| Yes, these three were best \| \| --- \| --- \| \| Ex. 1: The threat-drive and security system- Draw what you have in the circles  Ex. 2: Self-focused breathing – mindfulness exercise  Ex. 3: The train  Ex. 4: Try to understand yourself – fill in background, problems, strategies, etc.  Ex. 5: Healing ray of light – mindfulness exercise focused on tension/fatigue  Ex. 6: What do you value highly in your life? Choose 5 values.  Ex. 7: Create an image of compassion  Ex. 8: Draw a tree of life  Ex. 9: Personal goals regarding food, drink, sleep, activities  Ex. 10: Switch seats – imagine that you are talking to yourself with a friendly tone. \|  \|   7. Do you feel that the following exercises have helped you reach your goals (increase self-compassion, reduce self-criticism and increase affect-regulation abilities)?   \|  \| No, not at all \| Yes, a little \| Yes, sufficiently \| Yes, more than sufficiently \| Yes, to a large extent \| Did not do/No \| \| --- \| --- \| --- \| --- \| --- \| --- \| --- \| \| Ex. 1: Balloon breathing \|  \|  \|  \|  \|  \|  \| \| Ex. 2: Box breathing \|  \|  \|  \|  \|  \|  \| \| Ex. 3: Body control – stand up and close your eyes and imagine a push. \|  \|  \|  \|  \|  \|  \| \| Ex. 4: Analyze your feeling – vulnerability, trigger, emotion, thought, behavior, etc. \|  \|  \|  \|  \|  \|  \| \| Ex. 5: Mountain and stable – mindfulness exercise \|  \|  \|  \|  \|  \|  \| \| Ex. 6: Identify thought traps and create alternative thoughts \|  \|  \|  \|  \|  \|  \| \| Ex. 7: How is your tone? \|  \|  \|  \|  \|  \|  \| \| Ex. 8: Safe place \|  \|  \|  \|  \|  \|  \| \| Ex. 9: Mind map \|  \|  \|  \|  \|  \|  \| \| Ex. 10: Imagination and music. Think about something stressful, listen to music, relax. \|  \|  \|  \|  \|  \|  \| |

8. Rate the exercises. Mark only three (3) of the exercises – the exercises that you think most helped you reach your goals (increase self-compassion, reduce self-criticism and increase affect-regulation abilities).

|  | Yes, these three were best |
| --- | --- |
| Ex. 1: Balloon breathing |  |
| Ex. 2: Box breathing |  |
| Ex. 3: Body control – stand up and close your eyes and imagine a push. |  |
| Ex. 4: Analyze your feeling – vulnerability, trigger, emotion, thought, behavior, etc. |  |
| Ex. 5: Mountain and stable – mindfulness exercise |  |
| Ex. 6: Identify thought traps and create alternative thoughts |  |
| Ex. 7: How is your tone? |  |
| Ex. 8: Safe place |  |
| Ex. 9: Mind map |  |
| Ex. 10: Imagination and music. Think about something stressful, listen to music, relax. |  |

9. How do you rate the level of the training/course in relation to your knowledge?

Too easy

Somewhat easy

Neither too easy nor too difficult

Rather difficult

Too difficult

10. How was your relationship with the group leaders?

1. I did not feel heard, understood or respected

2.

3.

4.

5. I felt heard, understood and respected

11. What we did in Compassion-Focused Training was important to me.

No, not at all

Yes, at times

Yes, somewhat

Yes, most of the time

Yes, always

12. If a friend was in need of similar help, would you recommend our CFT training to him or her?

No, definitely not

No, I don't think so

Yes, I think so

Yes, definitely

13. How satisfied are you with the extent (seven 1.5-hour meetings) of the help you received?

Far too few meetings

Neither satisfied nor dissatisfied

Mostly satisfied

Very satisfied

14. Has the training you received helped you better cope with your problems?

Yes, it has helped a lot

Yes, it has helped somewhat

No, it hasn’t really helped

No, it seems to have made it worse

15. Overall, how satisfied are you with the training you have received?

Very satisfied

Mostly satisfied

Neither satisfied nor dissatisfied

Dissatisfied
